# Supplementary material for: Patterns of Intron Gain and Loss in Fungi
Source: PLoS Biol. 2004 Nov 30;2(12):e422. doi: 10.1371/journal.pbio.0020422 (PMC532390; doi:10.1371/journal.pbio.0020422)
Supplement: Table S1 — Also available at http://genes.mit.edu/NielsenEtAl/. (4.3 MB ZIP). [file pbio.0020422.st001.zip › NielsenEtAl/html/72.html]

AN4208.1.NCU04144.1.MG06765.1.FG02016.1


```
 CLUSTAL W (1.82) Multiple Sequence Alignments - Introns Inserted


Sequence 1: NCU04144.1	580 aa
Sequence 2: FG02016.1	606 aa
Sequence 3: MG06765.1	594 aa
Sequence 4: AN4208.1	562 aa
Alignment Length: 652 aa
Number Identitical Residues: 253 aa
Alignment Score (without introns) 13387


MG06765.1 	MSTTSAELLNGANGRINDHDHVLRPRPRKPLHSQLS---QISNASDTGSTFDASSEHG-~
NCU04144.1	-MTS---TSTSADAFAGQGDNILRPRPRKPLHTQVS---QLSHDANGDSDHANGTGATS2
FG02016.1 	-MSSSIETLANGNGHAGEHDHVLRPRPRKPKHSQVSDVVRMTSTSDGEHLMPDNASTGS~
AN4208.1  	--------MSSAVSHQ-DGSAVLRPR-------------AVPDARKVEDVLLS-SQIEK~
          	          .. .   : . :****              :.   .        :   . 

MG06765.1 	-ASIS2S~GRSTPVPEDAPPSVKILSTARQQVRREQREILRSRRRLFPPTDFESRLSHLD
NCU04144.1	VTGVS~S~GRSTPIPSDAPPSIQSLSTARKQVRAEQR------RRIFPTIEFASRVSHFD
FG02016.1 	ASGLT~S2GRSTPVPENAPPSVKSLSTARKQVRAEQR------RRIFPTIEFASRVSHFD
AN4208.1  	LDTES~S2GHSTPIPEDAPPSAHSISTARKQARARNR--------LFYTIHYVPRVSHFD
          	    : * *:***:*.:**** : :****:*.* .:*        :* . .: .*:**:*

MG06765.1 	PNSDYRDFHGFFNLFWIGLAIMAITTMLRNIKDTGYPMRVQIWGLFTVKVWELALIDAAM
NCU04144.1	PNSDYRDFHGFFNLFWIGLAIMAITTCLRNIKDTGYPLRVQIWGLFTVKLWHLAIADFLM
FG02016.1 	PESDYRDFHGFFNLFWIGLAIMGITSMLRNLKDTGFPLRVQIWSLFTVKLWHLAIADFLM
AN4208.1  	PRSDYHNFRGFFTLFWIGLFIMVVTTVLRNIKDTGYPLRVRVWSLLTANVWSMGFSDIAM
          	*.***::*:***.****** ** :*: ***:****:*:**::*.*:*.::* :.: *  *

MG06765.1 	VLSTYVSLPLHRVFRAAPAGSLLTWARGGMAIMSIYEVLWLALWVA~TPFLLDWTWTAQV
NCU04144.1	VATTAVSLPLHKVFRAAPAGGVLTWAKGGMAIQSIYQAIWLAVWIV~IPFWLEWTWTAQV
FG02016.1 	VATTAVSLPLNRAARNAKPGSFLDWNKGGIAIQSIYQVVWLTLWII~VPFYFEWTWTSQV
AN4208.1  	VCTSGLVIPLQYLSRTNGP---FRWERGGIVLQSIFEVVWLGVWIK2WPFMLRWTWTAQV
          	* :: : :**:   *   .   : * :**:.: **::.:** :*:   ** : ****:**

MG06765.1 	FLLLHTMVLLMKMHSYVFYNGHLSETEKRLRALDNPSTAS-KAPAYLYPSENKVASPKRT
NCU04144.1	FLLLHTMVLLMKMHSYSFYMGHLSETEKRLRALDDPATAS-KAPAYLYPTPENPLGTVAS
FG02016.1 	FFLLHTMVLLMKMHSYAFYNGHLSQTEKRLHALDNPFSPHDRRPAYQYPSTDLPASVTKG
AN4208.1  	FFTLHTLTILMKIHSYAFYNGHLSATERRLSELDKPGSQP-TGEAVRYPEVPTSKQPLRR
          	*: ***:.:***:*** ** **** **:**  **.* :      *  **           

MG06765.1 	TAP-----KFFRDNKADETSSPLETSTLSKDVDDRVGG------DWHVDGEDDDISQLRE
NCU04144.1	P---------------KQRAVEAAAEASSKETEEI-----------STPNEDEEITQLRE
FG02016.1 	ESNSTNAKKRRRQRQKKQEAKQSANGHKSNDEEPINHDLLAPSPVASPSNSADEVDEIRE
AN4208.1  	P---------------------------------------------SIHKRSNSVAQLRD
          	                                                    :.: ::*:

MG06765.1 	DLARELTSPMGNTAYPRNLTFSNYVDYLFCPTLCYELEYPRTEKIDWQSLISKIIAVFGC
NCU04144.1	DLARELTSPMGNITYPANLTWANYLDYLCCPTLCYEIEYPRTAAIDWQNLLSKVLAVFGC
FG02016.1 	DLARELTSPIGNVTYPNNLTWSNYTDYIFCPTLCYEIEYPRTTHINWINLISKIIATFGC
AN4208.1  	DLATELTSPMGNVTYPQNLTLYNYVDFIFCPTLCYELEYPRSKERSWMEVGYKTLAVFGC
          	*** *****:** :** ***  ** *:: *******:****:   .* .:  * :*.***

MG06765.1 	IFLLTIVSEEFILPVLIESSQRLA-----VPGMS----ASETILILCETISWLLFPFMVT
NCU04144.1	IFLLTVISEEFILPALTDASIRLDPTLRAAAGDSGPLTALEALLVLAETISWLLFPFMLT
FG02016.1 	IFLLTIISEDFILPVLLDASKRLD----AVPTES---SVSEKLLILAETISWLLFPFMLT
AN4208.1  	IFLLTLVSEEFILPVLAEAGVRLH-----AANTP-----SDKALIMAEAISMLLFPFMIT
          	*****::**:****.* ::. **      ..  .      :  *::.*:** ******:*

MG06765.1 	FLLVFLVIFEYVLGACAEMTRFADRRFFADWWNSTDW~MEFSREWNVPVHAFLRRHVYGA
NCU04144.1	FLLVFLVIFEYVLGAFAEITHFADRHFYSDWWNSTDW~MEFSREWNVPVYNFLRRHVYSA
FG02016.1 	FLLVFLVIFEYVLGAFAEITFFADRHFYSDWWNSTDW~MEFSREWNIPVYSFLRRHVYSA
AN4208.1  	FLLVFLVIFEYVLGAFAELTCFADRHFYSDWWNSCDW2LEFSREWNVPVHHFLRRHVYFS
          	*************** **:* ****:*::***** ** :*******:**: ******* :

MG06765.1 	SRPHIGKPYATVITFLISAIGHEIVMACITKKLRGYGLVCQMLQLPIFALQRTKWIRGKH
NCU04144.1	SRPHVGKANATVITFLISAVGHEIVMACITKKLRGYGFICQMLQLPIVMLQKTELVRGKK
FG02016.1 	SRPRIGRGAATALTFLISAFGHEIVMACITKKFRGYGFVCQMLQLPIVMLQRTRWVRGKE
AN4208.1  	SLSHFSKPVAMFITFLVSSIFHELVMSCITKKLRGYGFLAMMLQLPIIAIQKSKYFRHKT
          	* .:..:  *  :***:*:. **:**:*****:****::. ******. :*::. .* * 

MG06765.1 	TAN~NVFFWCSMIMGLSM0--------MCSLYVLI-----~-------------------
NCU04144.1	TLN~NVCFWCSMILGLSL0--------ICSLYVLV-----~-------------------
FG02016.1 	TLN~NVCFWASMVLGLSM0--------ICALYVLV-----~-------------------
AN4208.1  	TLN0NVFFWLSMIFGLSL0GAVDWSVLICCKIVFLVDLNL1ESYVYIFAHEKHSTNLNAK
          	* * ** ** **::***: .: . :  :*.  *:: . .  .:     : .. ::. .:.

NCU04144.1	
FG02016.1 	
MG06765.1 	
AN4208.1  	
          	
```
